# [4-(Methyl­sulfon­yl)phen­yl]acetic acid

**DOI:** 10.1107/S160053680803660X

**Published:** 2008-11-26

**Authors:** Yan-Qin Yuan, Sheng-Rong Guo, Li-Jin Wang

**Affiliations:** a Department of Chemistry, Lishui University, 323000 Lishui, Zhejiang Province, People’s Republic of China

## Abstract

In the crystal structure of the title compound, C_9_H_10_O_4_S, centrosymmetrically related mol­ecules are linked into dimers by inter­molecular O—H⋯O hydrogen bonds. Unconventional C—H⋯O hydrogen-bond inter­actions are also present, connecting dimers into a three-dimensional network.

## Related literature

For general background on the properties of the title compound and its derivatives, see: Parimalan *et al.* (2008 ▶); Giridhar *et al.* (2006 ▶). For the crystal structures of related compounds, see: Guo & Yuan (2006 ▶); Hartung *et al.* (2004 ▶); Hodgson & Asplund (1991 ▶). For hydrogen-bond motifs, see: Bernstein *et al.* (1995 ▶).
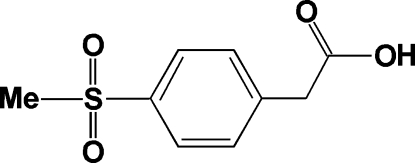


## Experimental

### 

#### Crystal data

C_9_H_10_O_4_S
*M* *_r_* = 214.23
Monoclinic, 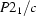

*a* = 19.086 (7) Å
*b* = 4.9711 (18) Å
*c* = 10.724 (4) Å
β = 106.102 (6)°
*V* = 977.5 (6) Å^3^
*Z* = 4
Mo *K*α radiation
μ = 0.32 mm^−1^
*T* = 298 (2) K
0.52 × 0.30 × 0.24 mm

#### Data collection

Bruker SMART APEX area-detector diffractometer
Absorption correction: multi-scan (*SADABS*; Sheldrick, 2004 ▶) *T*_min_ = 0.853, *T*_max_ = 0.928
4462 measured reflections
1638 independent reflections
1502 reflections with *I* > 2σ(*I*)
*R*_int_ = 0.019

#### Refinement

*R*[*F* ^2^ > 2σ(*F* ^2^)] = 0.048
*wR*(*F* ^2^) = 0.140
*S* = 1.11
1638 reflections
129 parameters
H-atom parameters constrained
Δρ_max_ = 0.24 e Å^−3^
Δρ_min_ = −0.43 e Å^−3^

### 

Data collection: *SMART* (Bruker, 2002 ▶); cell refinement: *SAINT* (Bruker, 2002 ▶); data reduction: *SAINT*; program(s) used to solve structure: *SHELXS97* (Sheldrick, 2008 ▶); program(s) used to refine structure: *SHELXL97* (Sheldrick, 2008 ▶); molecular graphics: *SHELXTL* (Sheldrick, 2008 ▶); software used to prepare material for publication: *SHELXL97*.

## Supplementary Material

Crystal structure: contains datablocks I, global. DOI: 10.1107/S160053680803660X/rz2263sup1.cif

Structure factors: contains datablocks I. DOI: 10.1107/S160053680803660X/rz2263Isup2.hkl

Additional supplementary materials:  crystallographic information; 3D view; checkCIF report

## Figures and Tables

**Table 1:** Hydrogen-bond geometry (Å, °)

*D*—H⋯*A*	*D*—H	H⋯*A*	*D*⋯*A*	*D*—H⋯*A*
O4—H4⋯O3^i^	0.82	1.87	2.693 (3)	175
C3—H3⋯O2^ii^	0.93	2.53	3.287 (3)	139
C1—H1*B*⋯O1^iii^	0.96	2.45	3.365 (4)	160

Symmetry codes: (i) 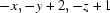; (ii) 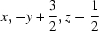; (iii) 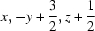.

**Fig. 1.**
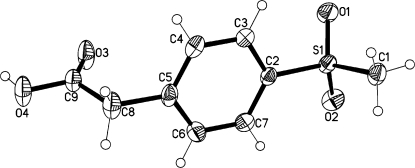
The molecular structure of the title compound, with the atom-labeling scheme and 50% probability displacement ellipsoids.

## supplementary crystallographic information

### Comment

The title compound, C_9_H_10_O_4_S, and its substituted derivatives have been found to possess an auxin-like activity (Parimalan *et al.*, 2008; Giridhar *et al.*, 2006). These compounds, which are predominantly found in fruits, can be used in the synthesis of pharmaceutical intermediates, some perfumes and non-steroidal anti-inflammatory drugs.

In the molecule of the title compound (Fig. 1), bond lengths and angles agree well with those observed in similar compounds (Guo & Yuan, 2006; Hartung *et al.*, 2004; Hodgson & Asplund, 1991). The S═O bond lengths within the SO_2_Me group are not significantly different, with an average value of 1.4366 (10) Å. The average bond length for the two C—S bonds is 1.762 (3) Å. In the crystal packing, centrosymmetrically related molecules are linked into dimers by intermolecular O—H···O hydrogen bonds (Table 1) generating an eight-membered ring of graph set *R*^2^_2_(8) (Bernstein *et al.*, 1995). The dimers are further linked into a three-dimensional network by unconventional C—H···O hydrogen bonding interactions. The corresponding phenylacetic acid derive without the SO_2_Me group (Hartung *et al.*, 2004) forms helical columns of single enantiomers linked by hydrogen bonds between the acidic proton of one molecule and the methoxy O atom of a neighbouring molecule, to give an overall racemic structure.

### Experimental

1-(4-Methanesulfonyl-phenyl)-ethanone (20 mmol), morpholine (60 mmol) and elemental sulfur (40 mmol) were added in a round-bottom flask and refluxed for 2 h at 398 K. A 3N solution of NaOH (20 ml) was then added, and the reaction mixture refluxed for an additional 30 min. After cooling, the mixture was filtered and the filtrate was acidified with HCl to pH 6. The solution was again filtered off and washed with ethyl acetate. The resulting aqueous fraction was finally acidified with diluted HCl, to yield the pure product as a white solid. Crystals suitable for X-ray analysis were obtained by slow evaporation of an ethanol/water (1:1 v/v) solution.

### Refinement

All H atoms were placed at calculated positions and constrained to ride on their parent atoms, with O—H = 0.82 Å, C—H = 0.93-0.97 Å and with *U*_iso_(H) = 1.2 *U*_eq_(C) or 1.5 *U*_eq_(C, O) for hydroxy and methyl H atoms.

### Crystal data

**Table:**

C_9_H_10_O_4_S	*F*_000_ = 448
*M**_r_* = 214.23	*D*_x_ = 1.456 Mg m^−^^3^
Monoclinic, *P*2_1_/*c*	Mo *K*α radiation λ = 0.71073 Å
Hall symbol: -P 2ybc	Cell parameters from 2824 reflections
*a* = 19.086 (7) Å	θ = 2.2–27.7º
*b* = 4.9711 (18) Å	µ = 0.32 mm^−^^1^
*c* = 10.724 (4) Å	*T* = 298 (2) K
β = 106.102 (6)º	Block, colourless
*V* = 977.5 (6) Å^3^	0.52 × 0.30 × 0.24 mm
*Z* = 4	

### Data collection

**Table:**

Bruker APEX area-detector diffractometer	1638 independent reflections
Radiation source: fine-focus sealed tube	1502 reflections with *I* > 2σ(*I*)
Monochromator: graphite	*R*_int_ = 0.020
*T* = 298(2) K	θ_max_ = 25.0º
φ and ω scans	θ_min_ = 1.1º
Absorption correction: Multi-scan(SADABS; Sheldrick, 2004)	*h* = −22→22
*T*_min_ = 0.853, *T*_max_ = 0.928	*k* = −5→5
4462 measured reflections	*l* = −11→12

### Refinement

**Table:**

Refinement on *F*^2^	Secondary atom site location: difference Fourier map
Least-squares matrix: full	Hydrogen site location: inferred from neighbouring sites
*R*[*F*^2^ > 2σ(*F*^2^)] = 0.048	H-atom parameters constrained
*wR*(*F*^2^) = 0.140	*w* = 1/[σ^2^(*F*_o_^2^) + (0.0848*P*)^2^ + 0.3788*P*] where *P* = (*F*_o_^2^ + 2*F*_c_^2^)/3
*S* = 1.11	(Δ/σ)_max_ = 0.001
1638 reflections	Δρ_max_ = 0.24 e Å^−^^3^
129 parameters	Δρ_min_ = −0.43 e Å^−^^3^
Primary atom site location: structure-invariant direct methods	Extinction correction: none

### Special details

**Table:**

Geometry. All e.s.d.'s (except the e.s.d. in the dihedral angle between two l.s. planes) are estimated using the full covariance matrix. The cell e.s.d.'s are taken into account individually in the estimation of e.s.d.'s in distances, angles and torsion angles; correlations between e.s.d.'s in cell parameters are only used when they are defined by crystal symmetry. An approximate (isotropic) treatment of cell e.s.d.'s is used for estimating e.s.d.'s involving l.s. planes.
Refinement. Refinement of *F*^2^ against ALL reflections. The weighted *R*-factor *wR* and goodness of fit *S* are based on *F*^2^, conventional *R*-factors *R* are based on *F*, with *F* set to zero for negative *F*^2^. The threshold expression of *F*^2^ > σ(*F*^2^) is used only for calculating *R*-factors(gt) *etc*. and is not relevant to the choice of reflections for refinement. *R*-factors based on *F*^2^ are statistically about twice as large as those based on *F*, and *R*- factors based on ALL data will be even larger.

### Fractional atomic coordinates and isotropic or equivalent isotropic displacement parameters (Å^2^)

**Table:**

	*x*	*y*	*z*	*U*_iso_*/*U*_eq_	
S1	0.38734 (3)	0.64558 (12)	1.01463 (5)	0.0408 (3)	
O1	0.41630 (11)	0.5716 (4)	0.90951 (18)	0.0609 (6)	
O2	0.36962 (11)	0.4358 (4)	1.09321 (19)	0.0575 (5)	
O3	0.07977 (10)	0.9489 (5)	0.6087 (2)	0.0694 (7)	
O4	0.00360 (10)	1.2830 (5)	0.6090 (2)	0.0677 (6)	
H4	−0.0197	1.2097	0.5418	0.102*	
C1	0.44823 (14)	0.8670 (5)	1.1176 (3)	0.0487 (6)	
H1A	0.4947	0.7807	1.1501	0.073*	
H1B	0.4296	0.9159	1.1889	0.073*	
H1C	0.4537	1.0256	1.0701	0.073*	
C2	0.30708 (12)	0.8358 (4)	0.9483 (2)	0.0361 (5)	
C3	0.30404 (13)	1.0060 (5)	0.8448 (2)	0.0425 (6)	
H3	0.3440	1.0226	0.8117	0.051*	
C4	0.24133 (14)	1.1507 (5)	0.7913 (2)	0.0481 (6)	
H4A	0.2393	1.2662	0.7221	0.058*	
C5	0.18090 (13)	1.1260 (5)	0.8396 (2)	0.0434 (6)	
C6	0.18554 (13)	0.9570 (6)	0.9437 (3)	0.0491 (6)	
H6	0.1458	0.9413	0.9775	0.059*	
C7	0.24791 (13)	0.8111 (5)	0.9986 (2)	0.0457 (6)	
H7	0.2502	0.6975	1.0685	0.055*	
C8	0.11162 (14)	1.2792 (6)	0.7781 (3)	0.0571 (7)	
H8A	0.0838	1.2955	0.8410	0.069*	
H8B	0.1244	1.4594	0.7574	0.069*	
C9	0.06426 (13)	1.1525 (5)	0.6574 (3)	0.0451 (6)	

### Atomic displacement parameters (Å^2^)

**Table:**

	*U*^11^	*U*^22^	*U*^33^	*U*^12^	*U*^13^	*U*^23^
S1	0.0481 (4)	0.0392 (4)	0.0331 (4)	0.0129 (2)	0.0079 (3)	−0.0027 (2)
O1	0.0635 (11)	0.0752 (13)	0.0449 (11)	0.0256 (10)	0.0164 (9)	−0.0110 (10)
O2	0.0767 (13)	0.0387 (10)	0.0564 (12)	0.0120 (9)	0.0176 (10)	0.0082 (8)
O3	0.0530 (11)	0.0703 (14)	0.0648 (13)	0.0194 (10)	−0.0170 (9)	−0.0235 (11)
O4	0.0509 (11)	0.0665 (13)	0.0667 (14)	0.0203 (10)	−0.0155 (9)	−0.0117 (11)
C1	0.0470 (13)	0.0516 (15)	0.0415 (14)	0.0117 (11)	0.0024 (11)	−0.0031 (11)
C2	0.0381 (11)	0.0374 (12)	0.0294 (11)	0.0036 (9)	0.0038 (9)	−0.0038 (9)
C3	0.0420 (12)	0.0509 (14)	0.0338 (13)	0.0017 (10)	0.0090 (10)	0.0022 (10)
C4	0.0541 (14)	0.0492 (15)	0.0349 (13)	0.0037 (11)	0.0022 (11)	0.0066 (10)
C5	0.0387 (12)	0.0450 (14)	0.0378 (13)	0.0031 (10)	−0.0038 (10)	−0.0110 (10)
C6	0.0386 (12)	0.0585 (15)	0.0499 (15)	0.0000 (11)	0.0116 (11)	−0.0038 (13)
C7	0.0486 (13)	0.0489 (14)	0.0401 (14)	0.0013 (11)	0.0132 (11)	0.0062 (11)
C8	0.0487 (14)	0.0545 (16)	0.0557 (17)	0.0130 (13)	−0.0061 (12)	−0.0132 (13)
C9	0.0364 (12)	0.0471 (15)	0.0449 (14)	0.0044 (10)	−0.0001 (10)	0.0002 (11)

### Geometric parameters (Å, °)

**Table:**

S1—O1	1.4342 (19)	C3—C4	1.378 (4)
S1—O2	1.439 (2)	C3—H3	0.9300
S1—C1	1.752 (3)	C4—C5	1.394 (4)
S1—C2	1.773 (2)	C4—H4A	0.9300
O3—C9	1.212 (3)	C5—C6	1.380 (4)
O4—C9	1.303 (3)	C5—C8	1.510 (3)
O4—H4	0.8200	C6—C7	1.379 (4)
C1—H1A	0.9600	C6—H6	0.9300
C1—H1B	0.9600	C7—H7	0.9300
C1—H1C	0.9600	C8—C9	1.497 (4)
C2—C3	1.384 (3)	C8—H8A	0.9700
C2—C7	1.386 (3)	C8—H8B	0.9700
			
O1—S1—O2	118.61 (12)	C3—C4—H4A	119.6
O1—S1—C1	108.87 (13)	C5—C4—H4A	119.6
O2—S1—C1	107.99 (13)	C6—C5—C4	118.7 (2)
O1—S1—C2	107.44 (11)	C6—C5—C8	120.8 (2)
O2—S1—C2	107.72 (12)	C4—C5—C8	120.5 (2)
C1—S1—C2	105.44 (11)	C7—C6—C5	121.3 (2)
C9—O4—H4	109.5	C7—C6—H6	119.3
S1—C1—H1A	109.5	C5—C6—H6	119.3
S1—C1—H1B	109.5	C6—C7—C2	119.2 (2)
H1A—C1—H1B	109.5	C6—C7—H7	120.4
S1—C1—H1C	109.5	C2—C7—H7	120.4
H1A—C1—H1C	109.5	C9—C8—C5	114.3 (2)
H1B—C1—H1C	109.5	C9—C8—H8A	108.7
C3—C2—C7	120.6 (2)	C5—C8—H8A	108.7
C3—C2—S1	119.18 (17)	C9—C8—H8B	108.7
C7—C2—S1	120.22 (18)	C5—C8—H8B	108.7
C4—C3—C2	119.5 (2)	H8A—C8—H8B	107.6
C4—C3—H3	120.3	O3—C9—O4	122.7 (2)
C2—C3—H3	120.3	O3—C9—C8	124.2 (2)
C3—C4—C5	120.8 (2)	O4—C9—C8	113.0 (2)
			
O1—S1—C2—C3	−35.3 (2)	C3—C4—C5—C8	−178.3 (2)
O2—S1—C2—C3	−164.15 (19)	C4—C5—C6—C7	−1.0 (4)
C1—S1—C2—C3	80.7 (2)	C8—C5—C6—C7	178.4 (2)
O1—S1—C2—C7	143.9 (2)	C5—C6—C7—C2	0.2 (4)
O2—S1—C2—C7	15.0 (2)	C3—C2—C7—C6	0.5 (4)
C1—S1—C2—C7	−100.1 (2)	S1—C2—C7—C6	−178.71 (19)
C7—C2—C3—C4	−0.3 (4)	C6—C5—C8—C9	−98.9 (3)
S1—C2—C3—C4	178.84 (19)	C4—C5—C8—C9	80.5 (3)
C2—C3—C4—C5	−0.4 (4)	C5—C8—C9—O3	−2.1 (4)
C3—C4—C5—C6	1.1 (4)	C5—C8—C9—O4	177.6 (3)

### Hydrogen-bond geometry (Å, °)

**Table:**

*D*—H···*A*	*D*—H	H···*A*	*D*···*A*	*D*—H···*A*
O4—H4···O3^i^	0.82	1.87	2.693 (3)	175
C3—H3···O2^ii^	0.93	2.53	3.287 (3)	139
C1—H1B···O1^iii^	0.96	2.45	3.365 (4)	160

Symmetry codes: (i) −*x*, −*y*+2, −*z*+1; (ii) *x*, −*y*+3/2, *z*−1/2; (iii) *x*, −*y*+3/2, *z*+1/2.
